# Mortality, complication risks, and clinical outcomes after surgical treatment of spinal epidural abscess: a comparative analysis of patients aged 18–64 years, 65–79 years, and ≥ 80 years, with a 3-year follow-up

**DOI:** 10.1007/s10143-023-02003-6

**Published:** 2023-04-26

**Authors:** Pavlina Lenga, Gelo Gülec, Karl Kiening, Andreas W. Unterberg, Basem Ishak

**Affiliations:** Department of Neurosurgery, Heidelberg University Hospital, Im Neuenheimer Feld 400, 69120 Heidelberg, Germany

**Keywords:** Spinal epidural abscess, Surgery, Comorbidities, Intravenous drug abuse

## Abstract

Spinal epidural abscess (SEA) with pyogenic vertebral osteomyelitis (PVO) is a rare illness with a steadily increasing incidence. However, comparative analyses of young and older patients with SEA are lacking. We aimed to compare the clinical course of patients aged 18–64 years, 65–79 years, and ≥ 80 years undergoing surgery for SEA. Clinical and imaging data were retrospectively collected from the institutional database between September 2005 and December 2021. Ninety-nine patients aged 18–64 years, 45 patients aged 65–79 years, and 32 patients ≥ 80 years were enrolled. Patients ≥ 80 years presented with a poorer baseline history (9.2 ± 2.4), as indicated by the CCI, than their younger counterparts (18–74 years: 4.8 ± 1.6;6.5 ± 2.5; p < 0.001). Patients aged 65–79 years and 80 years had a significantly longer length of stay. In-hospital mortality was significantly higher in those aged ≥ 80 years compared to their younger counterparts (≥ 80 years, n = 3, 9.4% vs. 18–64 years, n = 0, 0.0%; 65–79 years, n = 0, 0.0%; p < 0.001), while no differences in 90-day mortality or 30-day readmission were observed. After surgery, a significant decrease in C-reactive protein levels and leukocytes and amelioration of motor scores were observed in all the groups. Of note, older age (> 65 years), presence of comorbidities, and poor preoperative neurological condition were significant predictors of mortality. Surgical management led to significant improvements in laboratory and clinical parameters in all age groups. However, older patients are prone to multiple risks, requiring meticulous evaluation before surgery. Nevertheless, the risk profile of younger patients should not be underestimated. The study has the limitations of a retrospective design and small sample size. Larger randomized studies are warranted to establish the guidelines for the optimal management of patients from every age group and to identify the patients who can benefit from solely conservative management.

## Introduction

Spinal epidural abscess (SEA) is a rare and devastating illness resulting from the accumulation of purulent fluid between the spinal dural mater and the vertebral periosteum. Currently, its incidence in developed countries is steadily rising and is estimated to range from 0.2 and 2 cases per 10.000 hospital admissions [1, 2]. This condition has attracted increasing attention given the increasing mortality rates for this serious disease, ranging from 15 to 23% [2–4]. Some of the potential contributing factors associated with the increasing prevalence of the disease include advanced age and concomitant comorbidities such as diabetes mellitus, renal failure, and immune suppression, as well as intravenous (IV) drug abuse, mainly in young groups [5].

SEA symptoms in the acute setting include progressive neurological decline and development of new motor deficits; hence, MRI appears to be important to confirm the diagnosis [6, 7]. Prompt surgical decompression and evacuation with concurrent antibiotic treatment seem to be state-of-the-art therapy for such a condition, especially in the presence of neurological deficits [5, 8]. Previous studies suggested that such therapeutic approaches might benefit older patients or even octogenarians by preserving or improving their neurological condition; nevertheless, a multidisciplinary approach and understanding of their unique needs are important aspects given their poor baseline reserve [3, 9]. However, there is a lack of comparative studies involving young and older patients with SEA focusing on the outcomes after surgical management. Owing to the lack of robust clinical evidence on this topic, this study sought to assess and compare the clinical course and determine the morbidity and mortality rates among patients with SEA aged 18–64 years, 65–79 years, and ≥ 80 years who underwent surgical treatment. We also assessed the potential risk factors for mortality, with a special emphasis on patient age.

## Methods

### Study design and patients’ characteristics

**Fig. 1:**
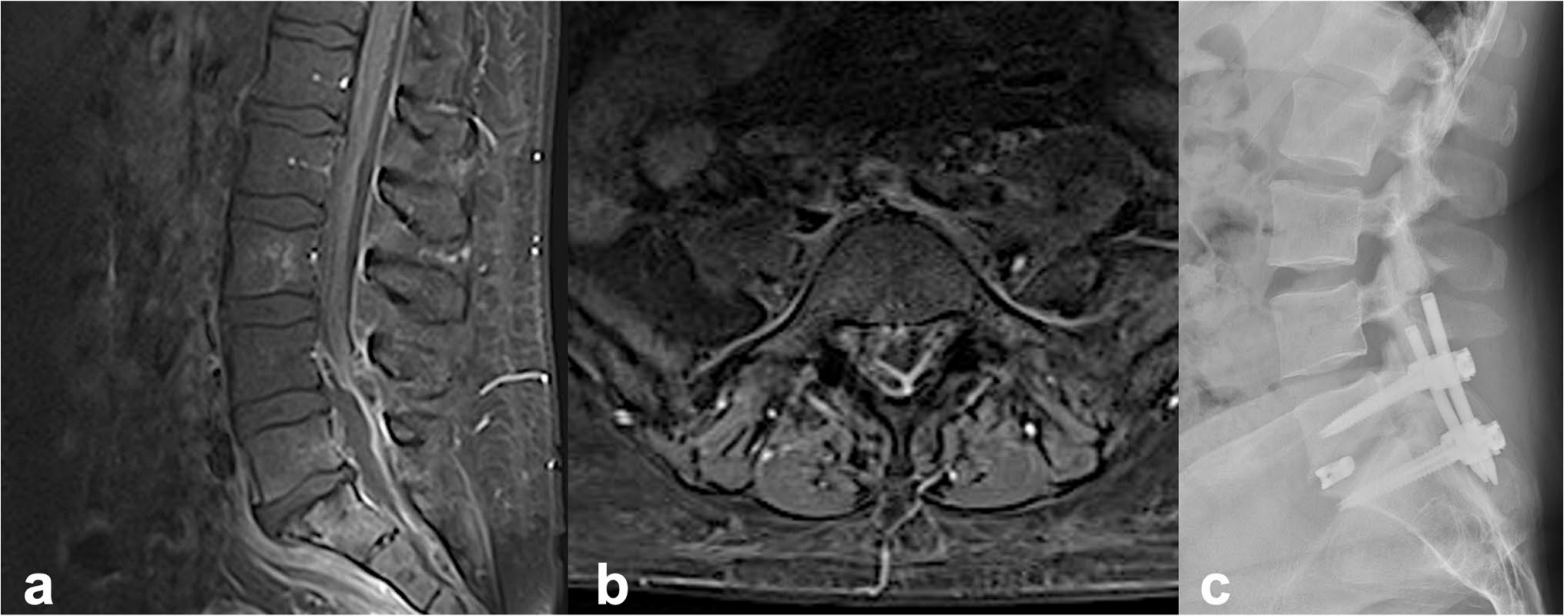
Postcontrast sagittal (**a**) and axial (**b**) magnetic resonance (T1 gadolinium sequence) imaging of dorsal lumbar epidural abscess and early end plate destruction of L5 and S1 of an 36-year-old male patient with intravenous drug abuse presenting with lumbar pain and progressive motor weakness of low extremeties. (**c**) Lateral radiographic view of posterior instrumentation and fusion extending from level L5 to S1

**Fig. 2:**
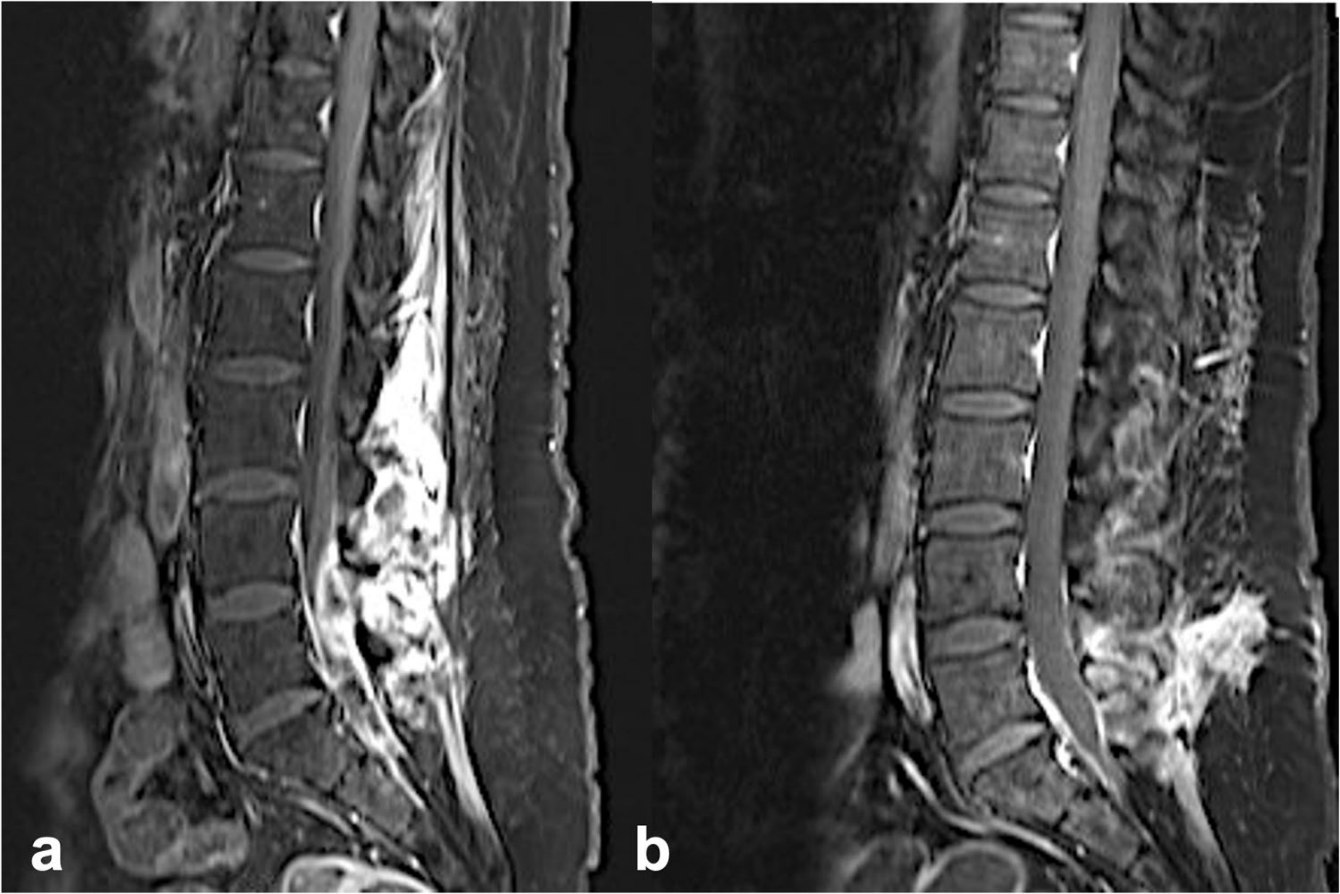
Postcontrast sagittal (**a**) (T1 gadolinium sequence) imaging of dorsal lumbar epidural abscess (L3-S1) of an 24-year-old female patient with alcohol and i.v. drug abuse presenting with back pain and progressive low extremety weakness. Postcontrast sagittal (**b**) (T1 gadolinium sequence) after 4 months follow up depicting a sufficient evacuation of the epidural abscess

Both clinical and imaging data were retrospectively collected between September 2005 and December 2021) from our institutional database. This study was conducted in accordance with the Declaration of Helsinki. The local ethics committee approved the study (approval number 880/2021). The requirement for informed consent was waived because of the retrospective nature of the study. Patients aged 18–64 years, 65–79 years, and ≥ 80 years with SEA with pyogenic vertebral osteomyelitis (PVO) across the thoracic and lumbar spine were consecutively enrolled. The diagnosis was based on magnetic resonance imaging (MRI) data (Figs. 1 and 2). The spinal stability of the affected spine was evaluated using computed tomography (CT). Osteoporotic signs were identified by the degree of vertebral reduction, cortical disruption, and impaction of trabeculae, with increased density adjacent to the endplate, as displayed in the CT scans. All patients aged ≥ 80 years presented with signs of osteoporosis. Bone densitometry was not possible because of the acute neurological deterioration demanding a swift switch to therapy. The exclusion criteria were as follows: bony deconstruction resulting in kyphosis or subluxation of the vertebral column, a vertebral collapse of > 50%, bone necrosis, complete loss of disc height, intracranial or cervical pathology, age < 18 years, and insufficient documentation of the requisite data.

### Demographic data and baseline characteristics

As already shown in our previous studies on SEA [3, 9], patient demographics, comorbidities, American Society of Anesthesiologists (ASA) scores, surgery duration, blood loss, number of treated spinal levels, perioperative and postoperative complications, hospital length of stay (LOS), intensive care unit (ICU) stay, readmission rate, reoperation rate, and mortality rate were retrieved from the patients’ electronic medical records. The age-adjusted Charlson comorbidity index (CCI) was used to assess the comorbidities [10, 11]. The CCI was calculated for each patient, and the scores were classified as follows: no comorbidity (CCI = 0), minimal comorbidity (CCI = 1 or 2), moderate comorbidity (CCI = 3–5), and severe comorbidity (CCI > 5). Pre- and post-surgical neurological status were assessed using the motor score (MS) of the American Spinal Injury Association (ASIA) impairment grading system (MS = 0, no muscle strength; MS = 100, healthy). Post-treatment MS data were obtained from the last documented clinical encounter. According to our institutional standards, routine clinical and radiological follow-up examinations were performed before discharge and 3 months after surgery. The follow-up period ranged between 3 months and 43.5 months after surgery. Conventional radiographs in the anterior–posterior and lateral views were obtained to evaluate the screw position and occurrence of fusion in patients who underwent spinal instrumentation. Follow-up MRI was performed only when there was a clinical suspicion of infection recurrence.

### Procedures

Patients were allocated to one of the following three age groups: 1) 18–64 years, 2) 65–79 years, and 3) ≥ 80 years. Since there is no consensus on a standardized therapy for managing SEA with PVO, treating the abscess was the first-line treatment. When the abscess was dorsally accessible, minimally invasive drainage via laminotomy was preferred, while for abscesses located ventrally in granulated tissue, an instrumentation approach was considered, as previously described (Camino Willhuber et al., 2021).

The classification system proposed by Pola et al. [12] was applied additionally in this study. Accordingly, the abscesses were classified as Type 3.C., indicating an epidural abscess without signs of instability, suggesting the need for surgical decompression alone. Of note, the diagnostic power of the tool was limited due to the low inter- and intra-rater variability (moderate-to-substantial agreement, Fleiss κ value: 0.6–0.63) [12, 13]. The decision-making process was mainly guided by the current neurological status (MS), concomitant underlying pathologies, the extent of the pathology, the potential risk of secondary instability [14, 15], and the discretion of an experienced clinical team consisting of neurosurgeons, neuroradiologists, and anesthesiologists. The attending spine surgeons made the final decision after carefully considering the aforementioned points. CT-based point-to-point navigation systems have been used for spinal instrumentation, as previously described by our study group [16]. In line with our institutional treatment protocol, blood samples or intraoperative cultures were first collected; then, IV antibiotics were initiated immediately. After identifying the bacterial specimens, specific IV antibiotics were administered. Vancomycin and meropenem were administered intravenously until the identification of the pathologic specimen, as previously suggested [17, 18]

#### ICU admission and hospital discharge

The decision making concerning the ICU admission or hospital discharge was based on our internal institutional standards considering the unique needs of each patient.

The decision to admit patients to ICU was determined by the severity of their illness. Severity of illness was defined by the magnitude of the acute disease, patient’s physical condition, and concurrent level of treatment and organ system support. Unambiguously, age served as a potential confounder when deciding patient admission to intensive care because of the poor preoperative baseline reserve.

Hospital discharge was based on the following parameters: patient cognitive status, patient activity level and function, suitability of patient’s current home, availability of family or caretaker support, ability to obtain medication, and availability of transportation from home for follow-up visits. If physicians deemed the discharge home was not suitable for the patient’s condition, the patient was referred to a rehabilitation clinic or nursing home.

### Statistical analysis

Continuous variables were presented as mean ± standard deviations and verified as being normally distributed using the Shapiro–Wilk test. Categorical variables were presented as numbers and percentages. Univariate analysis was used to compare group-wise baseline and surgical characteristics. Categorical variables were tested using the chi-squared test, whereas the independent t-test was used for continuous variables. The Kruskal–Wallis test was used to evaluate changes in C-reactive protein (CRP) levels, leukocyte count, and neurological status (MS) of each group at discharge. In the second stage, a regression analysis was conducted to identify the potential risk factors for mortality. Statistical significance was set at p < 0.05. All statistical analyses were performed using the Statistical Package for the Social Sciences software, version 24.0.0.0 (IBM, Armonk, NY, USA).

## Results

### Patient demographics

Over a period of 16 years, 99 patients aged 18–64 years, 45 aged 65–79 years, and 32 patients ≥ 80 years who were diagnosed with PVO and SEA were included. The overall mean age was 70.0 ± 3.2 years, with a predominance of the male sex (n = 118/181, 65.2%). Patients ≥ 80 years presented with a poorer baseline history (9.2 ± 2.4), as indicated by CCI, than their younger counterparts (18–74 years: 4.8 ± 1.6; ≥ 80 years: 6.5 ± 2.5; p < 0.001). The prevalence rates of cardiovascular diseases, peripheral vascular diseases, diabetes mellitus type II, and renal and liver failure were significantly higher in patients aged 65–79 years and ≥ 80 years than in the younger group. Both IV drug and alcohol abuse were highly prevalent in the 18–64-year-old group. No significant differences were observed among the groups regarding the extent or location of SEA. The rates of laboratory infection parameters were higher in both older groups (65–79 years: 160.8 ± 61.7 mg/L, ≥ 80 years: 140.8 ± 5.7 mg/L vs. 18–64 years: 101.1 ± 55.6; p < 0.001); similarly, older patients had higher rates of motor impairment (65–79 years: 81.5 ± 15.7, ≥ 80 years: 88.6 ± 15.4 vs. 18–64 years: 89.5 ± 13.0; p = 0.013), as evidenced by the MS score. The baseline characteristics are depicted in Table 1.

**Table 1:** Baseline patient characteristics

	18–64 y n = 99	65–79 y n = 49	≥ 80 y n = 32	p-value
Age, y (mean, SD)	54.8 (4.3)	72.6 (4.6)	82.6 (1.7)	** < 0.001**
Sex (n, %)				0.997
Male	62 (62.6)	35 (71.4)	21 (65.6)	
Female	37 (37.4)	14 (28.6)	14 (28.6)	
BMI, kg/m^2^ (mean, SD)	28.9 (7.8)	36.4 (8.9)	27.0 (4.5)	0.642
Comorbidities				
Age-adjusted CCI score (mean, SD)	4.8 (1.6)	6.5 (2.5)	9.2 (2.4)	** < 0.001**
Arterial hypertension (n, %)	34 (34.0)	29 (59.2)	26 (81.3)	** < 0.001**
Myocardial infarction (n, %)	9 (9.0)	20 (40.8)	19 (59.4)	** < 0.001**
Coronary heart disease (n, %)	18 (18.0)	21 (42.9)	19 (59.4)	0.456
Atrial fibrillation (n, %)	7 (7.0)	12 (24.5)	8 (25.0)	**0.010**
Heart failure (n, %)	0 (0.0)	7 (14.3)	9 (28.1)	**0.041**
Peripheral vascular disease	2 (2.0)	6 (12.2)	4(12.5)	0.087
COPD (n, %)	7 (7.0)	16 (32.7)	8 (25.0)	**0.032**
Diabetes mellitus Type II (n, %)	15 (15.0)	16 (32.7)	11 (34.4)	**0.004**
Renal failure (n, %)	14 (14.0)	7 (14.3)	16 (50.0)	**0.010**
Liver disease (n, %)	15 (15.0)	1 (3.1)	3 (6.1)	**0.051**
Gastrointestinal ulcer (n, %)	4 (4.0)	3 (6.1)	2 (6.3)	0.844
TIA/stroke (n, %)	3 (3.0)	6 (12.2)	2 (6.3)	0.303
Malignancy (n, %)	11 (11.0)	5 (10.2)	4 (12.5)	0.925
Dementia (n, %)	0 (0.0)	0 (0.0)	2 (6.3)	0.786
Alcohol abuse (n, %)	22 (22.0)	7 (11.9)	1 (3.1)	**0.006**
i.v. Drug abuse (n, %)	16 (16.2)	1 (1.7)	0 (0.0)	**0.026**
HIV (n, %)	8 (8.0)	3 (5.1)	0 (0.0)	0.251
Previous spinal surgery (n, %)	29 (29.0)	14 (28.6)	6 (18.8)	0.604
ASA class (n, %)				0.017
I	8 (8.1)	0 (0.0)	0 (0.0)	
II	44 (44.0)	15 (30.6)	5 (15.6)	
III	42 (42.0)	26 (53.1)	21 (65.6)	
IV	5 (5.0)	7 (14.3)	5 (15.6)	
V	0 (0.0)	1 (2.0)	1 (3.1)	
Localization of epidural abscess (n, %)				0.556
Thoracic	14 (14.1)	10 (20.4)	14 (43.8)	
Thoracolumbar	10 (10.0)	5 (10.2)	3 (9.4)	
Lumbar	65 (65.0)	24 (49.0)	12 (37.5)	
Lumbosacral	5 (5.0)	10 (20.4)	3 (9.4)	
CRP level, mg/L (mean, SD)	101.1 (55.6)	160.8 (61.7)	140.8 (91.8)	** < 0.001**
Leukocytes, count/L (mean, SD)	12.2 (5.9)	12.4 (6.3)	11.4 (5.7)	0.099
Preoperative MS score (mean, SD)	89.5 (13.0)	88.6 (15.4)	81.5 (15.7)	**0.014**

Bolded *p*-values indicate statistically significant results

*N *Group size; *n *Number of patients; *ASA *American Society of Anesthesiologists; *BMI *Body mass index; *CCI* Charlson comorbidity index; *COPD *Chronic obstructive pulmonary disease; *CRP* C-reactive protein; *MS* Motor score of the American Spinal Injury Association grading system; *SD* Standard deviation; *TIA* Transient ischemic attack

### Surgical procedures and clinical course

As shown in Table 2, no differences in surgical approach were observed among the groups. The surgical duration was significantly longer in patients aged 65–79 years and ≥ 80 years than in the younger group (≥ 80 years: 218.4 ± 112.0 min, 65–79 years: 176.5 ± 117.4 min vs. 18–64 years: 157.2 ± 118.1; p = 0.022). No significant differences were observed in the number of decompressed spinal levels among all three groups. Of note, patients aged 65–79 years and ≥ 80 years had a significantly higher LOS, while the length of ICU stay was similar between groups. In-hospital mortality was significantly higher those aged ≥ 80 years compared to their younger counterparts (18–64 years: n = 0, 0.0, ≥ 80 years: n = 3, 9.4% vs. 65–79 years: n = 0, 0.0%; p < 0.001), while no differences in 90-day mortality or 30-day readmission were observed. Laboratory parameter levels indicative of infection and motor impairment improved significantly quicker in younger patients. The overall mean follow-up period was 43.5 ± 12.1 months, and no reoperations due to secondary instability or deaths occurred. According to the radiographs, there were no cases of screw loosening or displacement. After surgery, a significant decrease in CRP levels and leukocyte count and amelioration of MS were observed in all groups at discharge when compared to the baseline levels, as displayed in Table 3.

**Table 2:** Comparison of surgical characteristics and clinical course among the groups

	18–64 y n = 99	65–79 y n = 49	≥ 80 y n = 32	p-value
Surgical approaches (n,%)				0.159
Laminectomy	46 (46.5)	28 (57.1)	18 (56.3)	
Instrumentation	53 (53.0)	21 (42.9)	14 (43.8)	
Intraoperative blood transfusion, n (%)	2 (2.0)	0 (0.0)	2 (4.1)	0.554
Surgical duration, min	157.2 (118.1)	176.5 (117.4)	218.4 (112.0)	**0.022**
No. of levels decompressed/fused	2.3 (0.9)	1.8 (1.0)	2.7 (1.2)	0.867
Hospital stay, days	12.9 (10.1)	11.6 (9.3)	23.3 (34.5)	**0.001**
ICU stay, days	4.6 (3.1)	3.5 (8.1)	1.8 (3.6)	0.130
Mortality				
In-hospital (n, %)	0 (0.0)	0 (0.0)	3 (9.4)	** < 0.001**
90-day (n, %)	15 (15.0)	3 (6.1)	4 (12.5)	0.278
30-day readmission, (n, %)	13 (13.0)	4 (8.1)	2 (6.2)	0.071
Post CRP	59.7 (45.1)	55.5 (47.1)	94.5 (70.9)	** < 0.001**
Post leukocyte count	8.9 (4.4)	8.8 (3.3)	9.9 (4.9)	0.198
Post MS	94.0 (11.4)	91.5 (10.2)	86.6 (13.0)	** < 0.001**

Except where otherwise indicated, quantities are mean (SD); **bold** = significant difference; Post, after surgery; Delta, difference between pre-and postsurgical values. *CRP* C-reactive protein; *ICU *Intensive care unit; *MS* Motor score of the American Spinal Injury Association grading system

**Table 3:** Occurrence of adverse events

	18–64 y n = 99	65–79 y n = 49	≥ 80 y n = 32	p-value
Deep wound infection	8 (8.1)	4 (8.2)	3 (9.4)	0.056
Acute heart failure	1 (1.0)	2 (4.1)	2 (6.3)	0.723
Thrombotic event	3 (3.0)	1 (2.0)	1 (3.1)	0.866
Septic shock	0 (0.0)	0 (0.0)	2 (6.3)	**0.007**
Pneumonia	0 (0.0)	0 (0.0)	7 (21.9)	** < 0.001**
Pleural effusion	0 (0.0)	6 (12.2)	2 (6.3)	0.602
Ileus	0 (0.0)	3 (6.1)	1 (3.1)	0.087
Urinary tract infection	0 (0.0)	2 (4.1)	1 (3.1)	0.186

All data are the number of patients (%)

Bolded *p*-values indicate statistically significant results

### Complications and risk factors for mortality

Pneumonia and septic shock occurred at a significantly higher rate in patients aged 65–79 years and ≥ 80 years (n = 7, 21.9%) than in younger patients (n = 0, 0.0%; p < 0.001). A detailed description of complications is provided in Table 4. The prevalence of infection with *Staphylococcus aureus* was high in all three groups (18–64 years: 65.0%, 65–79 years: 55.1%, and ≥ 80 years: 53.1%), either in the blood or intraoperative samples. Linear regression analysis showed that mortality was significantly associated with increased age (B = 4.1, p < 0.001). Binary logistic regression analysis revealed that older age (> 65 years), presence of comorbidities, and poor preoperative neurological condition were significant predictors for mortality, while the surgical approach itself and level of infection evidenced by blood samples were not (Table 5).

**Table 4:** Comparison between baseline (before surgery) and discharge

	18–64 y n = 99	18–64 y n = 99	p-value	65–79 y Baseline n = 49	65–79 y Discharge n = 49	p-value	≥ 80 y Baseline n = 32	≥ 80 y Discharge n = 32	p-value
CRP	101.1 (55.6)	59.7 (45.1)	** < 0.001**	160.8 (61.7)	55.5 (47.1)	** < 0.001**	140.8 (91.8)	94.5 (70.9)	**0.002**
Leukocytes	12.2 (5.9)	8.9 (4.4)	** < 0.001**	12.4 (6.3)	8.8 (3.3)	** < 0.001**	11.4 (5.7)	9.9 (4.9)	** < 0.001**
MS	89.5 (13.0)	94.0 (11.4)	** < 0.001**	88.6 (15.4)	91.5 (10.2)	**0.031**	81.5 (15.7)	86.6 (13.0)	**0.009**

All data are mean (SD)

Bolded *p*-values indicate statistically significant results

*CRP* C-reactive protein; *MS* Motor score of the American Spinal Injury Association grading system

**Table 5:** Risk factors associated with mortality

Risk factor	OR (95% CI)	p-value
Age > 65 years	1.2 (1.1–3.4)	**0.001**
Age-adjusted CCI score	1.8 (1.1–5.2)	**0.002**
Preoperative MS	1.7 (1.1–2.4)	**0.032**
Preoperative CRP	1.1 (1.0–1.8)	0.068
Duration of surgery	1.0 (0.9–1.3)	0.786
Number of levels decompressed	0.5 (0.1–1.0)	0.889
Surgical approach^b^	1.3 (1.1–2.1)	0.201

Bolded *p*-values indicate statistically significant results

*CCI *Charlson Comorbidity Index; *CI* Confidence interval; *ICU* Intensive care unit; *MS* Motor score of the American Spinal Injury Association grading system; *OR* Odds ratio; ^b^ posterior decompression and fusion

All octogenarians presented with osteoporotic signs as evaluated by CT images. We further performed a regression analysis exclusively for octogenarians to detect potential associations between osteoporosis and the occurrence of postoperative complications or mortality. No significant associations were found either between osteoporosis and mortality or osteoporosis and postoperative complications.

## Discussion

Although SEA is a rare entity, it represents a devastating illness with high morbidity and mortality rates and a high risk of neurological impairment, especially in case of a delayed diagnosis [2, 5, 8]. While surgical decompression and evacuation are considered the mainstays of treatment, the treatment outcomes and optimal therapy in light of patient age have not yet been clearly defined.

To the best of our knowledge, this is the first study to assess and compare the clinical characteristics and outcomes among patients aged 18–64 years, 65–79 years, and ≥ 80 years who underwent surgery for the management of SEA (decompression alone or decompression and instrumentation). We found that octogenarians had significantly higher rates of comorbidities, as indicated by the CCI, than the younger age groups, with cardiovascular diseases, renal failure, and diabetes mellitus being the most prevalent comorbidities. Interestingly, IV drug abuse and alcohol abuse were significantly more prevalent in the group aged 18–64 years. Older patients showed higher levels of infection markers in the blood, as evidenced by laboratory examination results, and worse neurological deficits than younger patients. Concerning the surgical characteristics, surgical duration was significantly longer in the oldest age group, while the surgical procedures, number of operated segments, and even intraoperative blood loss were similar among the three groups. As expected, octogenarians stayed longer in the hospital, while the younger patients showed quicker recovery from blood infections as well as neurological deficits. Octogenarians had a higher risk of complications such as pneumonia and septic shock. Older age, a larger number of underlying diseases, and poor preoperative neurological condition were significant risk factors for mortality, while the surgery itself or surgical duration was not.

The increasing life expectancy worldwide has led to a tremendous increase in the older population; therefore, the selection of treatments for various illnesses in this population has become a subject of debate. In fact, surgeons are reluctant to treat spinal conditions in older patients since increased age represents a poor prognostic factor mainly due to the poor baseline reserve [19].

Patients with immunocompromised status and those aged over 50 years are vulnerable to spinal infection and SEA, which are devasting illnesses [20, 21]. Shweikeh et al., in their retrospective analysis of SEA in 106 patients aged 33–89 years, reported that diabetes mellitus was the most prevalent comorbidity (38.9%), followed by cardiovascular disease (31.1%), renal failure (30%), and IV drug abuse (21.7%). Their results highlight that these underlying diseases should be meticulously considered when diagnosing SEA since these patient groups are predisposed to spinal infection [21]. In another study of 82 patients with SEA older than 50 years, the same comorbid diseases were mentioned as potential risk factors for the occurrence of SEA [22]. The largest meta-analysis of 915 patients with SEA highlighted that diabetes mellitus, renal failure, cardiovascular diseases, and IV drug abuse were the most important comorbid conditions that predisposed patients to spinal infections [2]. However, the aforementioned studies did not distinguish the baseline history of patients with respect to age and the potential impact of age at baseline on treatment outcomes, but they only reported on underlying conditions. For example, in their retrospective analysis comparing patients aged ≥ 65 years, Lenga et al. found that octogenarians had significantly higher frailty with a CCI of 9.2 than their younger counterparts with a CCI of 6.5, and the prevalence of renal failure was significantly higher in the octogenarian group. Akin to these findings, in their retrospective study of 16 older patients, Kim et al. identified the same comorbidities as predisposing factors for SEA [20]. In the present study, we compared the baseline characteristics of the patients with SEA according to the age group. As expected, younger patients had lower rates of comorbid diseases than the older group, but they showed the highest prevalence of IV drug abuse and alcohol abuse. In contrast older patients, aged 65–79 and ≥ 80 years, had a very poor baseline reserve with comorbidities of diabetes mellitus, renal failure, and cardiovascular diseases. Of note, older patients had more severe infections, as indicated by the CRP levels, than the younger patients. One potential explanation is that the aforementioned diseases lead to reduced immunocompetency; hence, the cellular immunity decreases, with reduced chemotaxis, phagocytosis, and bactericidal activity of neutrophilic granulocytes [23]. In addition, patients with chronic renal failure already have high levels of inflammatory markers in the blood due to the reduction in the number of T cells and cytokine levels; hence, a delayed or missed diagnosis might occur [24]. Therefore, when such patients visit the emergency unit, clinicians should be mindful of the fact that inflammatory processes are linked to an increased risk of spinal infection [25]. Regrading younger patients who abuse drugs, bacterial contamination of the equipment used for drug delivery and dysfunction of the cellular and humoral immune systems from chronic use of heroin could be the mechanisms underlying the development of SEA in this patient group [26].

Swift diagnosis and management of SEA are necessary to prevent the potentially devastating neurologic sequalae. In the present study, irrespective of the age group, all patients presented with acute neurological deficits, and in less than 24 h, surgical decompression with or without instrumentation and evacuation of SEA were performed to preserve or improve the neurological condition. Interestingly, our findings showed that older patients aged 65–79 years and octogenarians presented with worse neurological conditions than their younger counterparts. This phenomenon might be attributed to the fact that mechanical compression and vascular damage from hypoxia due to SEA might be more severe in older patients, given the high levels of infection markers in their blood, as described above [2, 5]. However, the surgical approach did not differ among the different age groups, and despite the surgical duration being longer in older patients, no significant differences were observed. It can be said that the longer surgical duration in older patients is due to the presence of degenerative changes that occur with aging. Nevertheless, older patients are usually frail, and surgical procedures come with high risks of peri- and postoperative complications. Notwithstanding, at our center, the patients were thoroughly evaluated, and considering the occurrence of new neurological deficits, surgical management of SEA seemed unavoidable. However, motor deficits and blood parameters improved significantly after emergency surgery across all three age groups. In line with these findings, Alton et al. reported a tremendous improvement in the neurological condition of the patients who underwent emergent surgery, with the postoperative MS decreasing drastically from 84.3 to 73.4. An improvement of the MS was seen in patients undergoing solely medical therapy with postoperative MS decreasing from 84.4 to 81.5; however recovery time lasted substantially longer [27]. In their study, the patients who presented with few motor deficits and low levels of infection markers in the blood were treated with antibiotic therapy. Of note, conservative therapy had failed in 18 patients, requiring surgical eradication of SEA [27]. In concordance with these findings, in their retrospective study of 77 patients aged 18–78 years, Connor et al. reported neurological improvement after surgery in approximately 80.0% of the patients [28]. Patel et al. stated that immediate surgery improves neurological conditions compared to medical therapy alone [8]. Nevertheless, the choice of medical or surgical intervention requires individual patient considerations, including age, concurrent conditions, and objective findings.

Of note, older patients had significantly longer hospital stays than their younger counterparts, possibly because the former have a poor baseline reserve along with a higher risk of postoperative complications, requiring close postoperative monitoring. The prevalence rates of pneumonia and septic shock were significantly higher in the older patients than in the younger ones, while a trend toward higher postoperative deep wound infection was seen in the younger age group. The in-hospital mortality was significantly higher in the octogenarian group, reaching 9.4%, while none of the patients died in the 65–79-year group, or in the young group. Of note, the deaths were not related to surgery but to the poor preoperative clinical status of the patients. The 90-day mortality was high in all three groups, ranging from 6.1% to 15.0%, but no significant differences were observed among the groups. In their analysis of patients with SEA, Shwekieh et al. did not divide the patients according to age; they reported an in-hospital mortality rate of 3.2%, which was much lower than the rates reported in our study. [21]. In another study of 163 patients with spinal infection, the 1-year mortality rate was 12.0%, akin to the rate described here [14]. In a large series based on claim data of patients with a mean age of 54 years who underwent surgery, the in-hospital mortality rate was 3%, while more than 26% of the sample experienced one or more complications. Interestingly, risk factors for mortality were increased age, motor deficits, and comorbid diseases such as renal failure or liver disease [29]. Similarly, we also found that increased age, presence of comorbidities, and poor preoperative neurological status were significant predictors of mortality, while the surgery itself did not have any impact on mortality. Considering all these points, a thorough discussion of the benefits and risks of the surgical procedure should be carried out with the patients and their family members since this illness is associated with hazardous complications, especially in older patients.

There is a notion that there might be a potential association between osteoporosis and the occurrence of infection [30]. Zhnag et al. stated that reduced bone mineral density might lead to infections, such as pneumonia, UTI, or even sepsis [30]. The underlying mechanism might be the reduced number of osteoblasts, which normally play a decisive role in lymphocyte production and thus in the immune defence. Owing to the decrease in their number, lymphopenia might occur. Hence, such patients are more prone to infections, and to postoperative complications.

Previous studies also described that patients suffering from osteoporotic fractures are at a higher risk for the development of spinal infection, as trauma-induced bone marrow edema and pseudoarthritis lead to the patient being bed ridden and chest expansion, resulting in higher risk of infection [31]. Considering the poor baseline reserve of octogenarians presenting with multiple comorbidities, this patient cohort may be susceptible to not only spinal infection but may also pose an undue risk of postoperative complications and mortality. However, according to our results, this theory could not be affirmed. This could be explained with the relatively small number of enrolled patients, which might have reduced the power of our regression analysis. This aspect warrants further research. Thus, prophylactic measures might be a potential key to mitigate postoperative complications in such a debilitating cohort.

In context of the current scenario, concerns are mounting regarding the safety profile of spinal instrumentation with additional fusion because of a potential spinal infection caused by the inserted material. In a retrospective study on 37 patients with spinal infection, Rayed et al. showed that solely instrumentation surgery was performed with success rates of over 80% with no implant failure [32]. Furthermore, Shomacher et al. confirmed that cages, irrespective of the material (titanium vs. polyetherketone), can be safely deployed in spinal infection cases with no risk of reinfection and comparable fusion rates [33]. In concordance with these findings, Talia et al. found a 0% rate of recurrence after spinal instrumentation with fusion [34]. The results of the current study affirm the previously reported findings, as we did not find any implant failure caused by infection recurrence or progression attributable to the implanted material. As depicted in Fig. 1, evacuation of the dorsally located abscess with concomitant instrumentation and fusion were performed. At the 3-month follow-up, the patient recovered completely with no clinical and laboratory signs of infection. Additionally, the implanted material was accurately positioned at the reconstruction site, as confirmed by the follow up X-rays. Thus, fusion in cases of spinal infection presents with a good safety profile as risks of infection recurrence or implant failure are approximately 0%.

At the 3-year follow-up, no revision surgery was needed in any of the groups, and no signs of secondary instability were observed. Previous studies have stated that radiologically confirmed secondary instability frequently occurs after spinal decompression, leading to the necessity for additional fusion surgery [35]. Therefore, even in the emergency setting, the spinal stability of the patients should be evaluated so that the need for additional surgeries and associated risks can be minimized as much as possible [36]. We believe that the meticulous preoperative examination of our cohort was a decisive factor that prevented revision surgery even in the frail patient group. Nevertheless, progressive degeneration of the spine and spontaneous fusion are frequently observed phenomena in older adults, and these conditions might cause instability [37].

### Clinical implications and outlook

Spinal infection is an increasing healthcare problem requiring prompt diagnosis and therapy to preserve the occurrence of hazardous complications. According to our findings, irrespective of the age group, in the presence of acute neurological deterioration, surgical treatment should be performed aiming to preserve the patient’s neurological status or to prevent further worsening. Concurrent antibiotic treatment tailored to the particular organism is mandatory to stop further progression of the infection. However, because older patients present with many comorbidities, such as renal failure, a meticulous adoption of medication doses should be considered. Herein, it is important to highlight that surgical management with concurrent antibiotic treatment led to substantial improvement of neurological status along all three age groups. Surgical management also did not differ between the groups. Overall, it seems that age may be a significant confounder when deciding for treatment, but even in severely affected patients with extensive infection and exceedingly high CRP levels, surgery should be initiated, if medically feasible. Because of lack of evidence-based criteria on optimal management of such patients, especially with respect to age, our study may serve as a basis for the development of guidelines and may be a decisive supportive tool for physicians who are reluctant to perform a surgical procedure because of the patients’ old age.

### Strengths and limitations

The main strength of the current study is that, to our knowledge, it is the first to investigate the outcomes of different age groups who underwent surgery for SEA. Our sample size was small, but since this patient group has been understudied so far, we think that our study provides a real-world picture of the disease and will help physicians in their decision-making. Second, the minimum follow-up period was 12 months; therefore, other relevant findings may not have been captured in our study. Third, selection bias may have been present because of the retrospective nature of the study. Associations between osteoporosis and the occurrence of postoperative complications and mortality could be seen. However, this aspect needs further research because according to the previous literature, osteoporosis might be a paramount factor for the higher rates of complications in such a cohort. Scores for admission to the ICU or hospital discharge were not assessed. However, an individualized concept with respect to the institutional standards was applied. Nevertheless, validated scores might be helpful tools for physicians to decide whether patients should be immediately admitted to the ICU or discharged home. Larger randomized studies are needed to identify potential candidates for non-operative management with antibiotic therapy only.

## Conclusions

Prompt diagnosis and emergency surgical evacuation of SEA seem to be the mainstay of treatment irrespective of patient age. However, older patients are prone to multiple risks, requiring meticulous evaluation before surgery. Nevertheless, the risk profile of younger patients should not be underestimated since drug and alcohol abuse, which were common in this age group, contribute to the reduction in their immune response. Furthermore, increased age, high CCI, and poor neurological status are critical factors that must be considered when selecting a therapeutic approach. A clear discussion with the patient and the relatives regarding the potential risk is strongly recommended.

## Data Availability

The datasets generated during and/or analyzed during the current study are available from the corresponding author on reasonable request.
